# Forced-air prewarming prevents hypothermia during living donor liver transplantation: a randomized controlled trial

**DOI:** 10.1038/s41598-022-23930-2

**Published:** 2023-04-06

**Authors:** Eun Jung Oh, Sangbin Han, Sooyeon Lee, Eun Ah Choi, Justin S. Ko, Mi Sook Gwak, Gaab Soo Kim

**Affiliations:** 1 Department of Anesthesiology and Pain Medicine, Samsung Medical Center, Sungkyunkwan University School of Medicine, 81 Irwon-ro, Gangnam-gu, Seoul, 06351 Korea; 2 Present Address: Department of Anesthesiology and Pain Medicine, Gwangmyeong Hospital, Chung-Ang University School of Medicine, Gwangmyeong, South Korea

**Keywords:** Medical research, Clinical trial design, Randomized controlled trials

## Abstract

Despite various intraoperative thermal strategies, core heat loss is considerable during liver transplantation and hypothermia is common. We tested whether forced-air prewarming prevents hypothermia during liver transplantation. Adult patients undergoing living donor liver transplantation were randomly assigned to non-prewarming group (*n* = 20) or prewarming group (*n* = 20). Patients in prewarming group underwent 30-min forced-air warming before anesthetic induction. During surgery, core temperature was measured in the pulmonary artery. The primary outcome was intraoperative hypothermia (< 36.0 °C). The secondary outcomes included plasma lactate concentration. Intraoperative hypothermia risk was significantly lower in prewarming group than in non-prewarming group (60.0% vs. 95.0%, *P* = 0.020). The difference in hypothermia incidence between groups was greater in the post-induction phase (20.0% vs. 85.0%, *P* < 0.001) than in the anhepatic or post-reperfusion phase, suggesting that prewarming mainly acts on preventing post-induction core-to-peripheral heat redistribution. Hypothermia duration was significantly shorter in prewarming group (60 [0–221] min vs. 383 [108–426] min, *P* = 0.001). Lactate concentration decreased during 3 h after graft reperfusion in prewarming group, whereas it continuously increased in non-prewarming group (− 0.19 [− 0.48 to 0.13] mmol/L vs. 1.17 [3.31–0.77] mmol/L, *P* = 0.034). In conclusion, forced-air prewarming decreases the incidence and duration of intraoperative hypothermia with potential clinical benefit while mainly acting by preventing the core-to-peripheral heat redistribution.

*Clinical trial registration*: Registered at the Clinical Research Information Service (https://cris.nih.go.kr, [KCT0003230]) on 01/10/2018.

## Introduction

Liver transplantation is attended with various intraoperative metabolic disturbances. Significant decrease in core temperature is one of them and results from decreased baseline functional reserve, extended surgical procedures, large exposure of intra-abdominal tissues, and the absence of hepatic heat production along with the insertion of cooled liver during the anhepatic phase^1^. Normally, core temperature is strictly controlled ranging from 36.5 to 37.5 °C to offer optimal thermal environment for various cells^2^. In contrast, hypothermic environment prevents optimal cell function and results in complications such as coagulopathy, immunomodulation, arrhythmia, and cardiac dysfunction as well as decreased tissue recovery from damage^3–8^. In particular, the newly transplanted liver graft is more vulnerable to any damages since it suffers from overload initiating rapid liver regeneration while performing full metabolic functions^9–11^. In this situation, hypothermia is thought to prevent from triggering various signals for active liver regeneration and increase the risk of graft failure^12^. Accordingly, intraoperative thermal managements have long been an issue in liver transplantation, and various thermal strategies have been introduced^13–15^. However, hypothermia is still not rare and the optimal thermal strategy remains unresolved.

Intraoperative core temperature drops dominantly immediately after the start of general anesthesia when the heat of the core compartment translates into the peripheral compartment due to dilatation of the peripheral vascular system which acts as the thermal barrier between the two compartments and controls the balance between core heat and peripheral heat^16^. This is not different in liver transplantation^15^; thus, it is important to prevent the core-to-peripheral heat redistribution during the post-induction phase to maintain intraoperative normothermia during liver transplantation. In this regard, previous studies in various surgical settings have suggested that active warming of peripheral tissues prior to anesthetic induction, which is the so-called prewarming, decreases the amount of the core-to-peripheral heat redistribution and prevents intraoperative hypothermia^18–19^ while forced-air prewarming has been widely accepted for its efficacy and safety^20^.

Because liver is the major thermoregulatory organ, liver transplant recipients may be more vulnerable to hypothermia induced by the core-to-peripheral heat distribution. Also, the vascular barrier to preserve core heat may be disturbed with severely decreased vascular resistance^21,22^. Nonetheless, the effects of prewarming have never been evaluated in liver transplantation while previous studies mainly focused on intraoperative warming after significant core heat loss already occured^13–15^. Thus, we hypothesized that prewarming decreases the amount of the core-to-peripheral heat redistribution and the risk of intraoperative hypothermia for liver transplant recipients. In this study, we tested whether forced-air prewarming prevents intraoperative hypothermia during liver transplantation.

## Materials and methods

### Subjects

**Figure 1:**
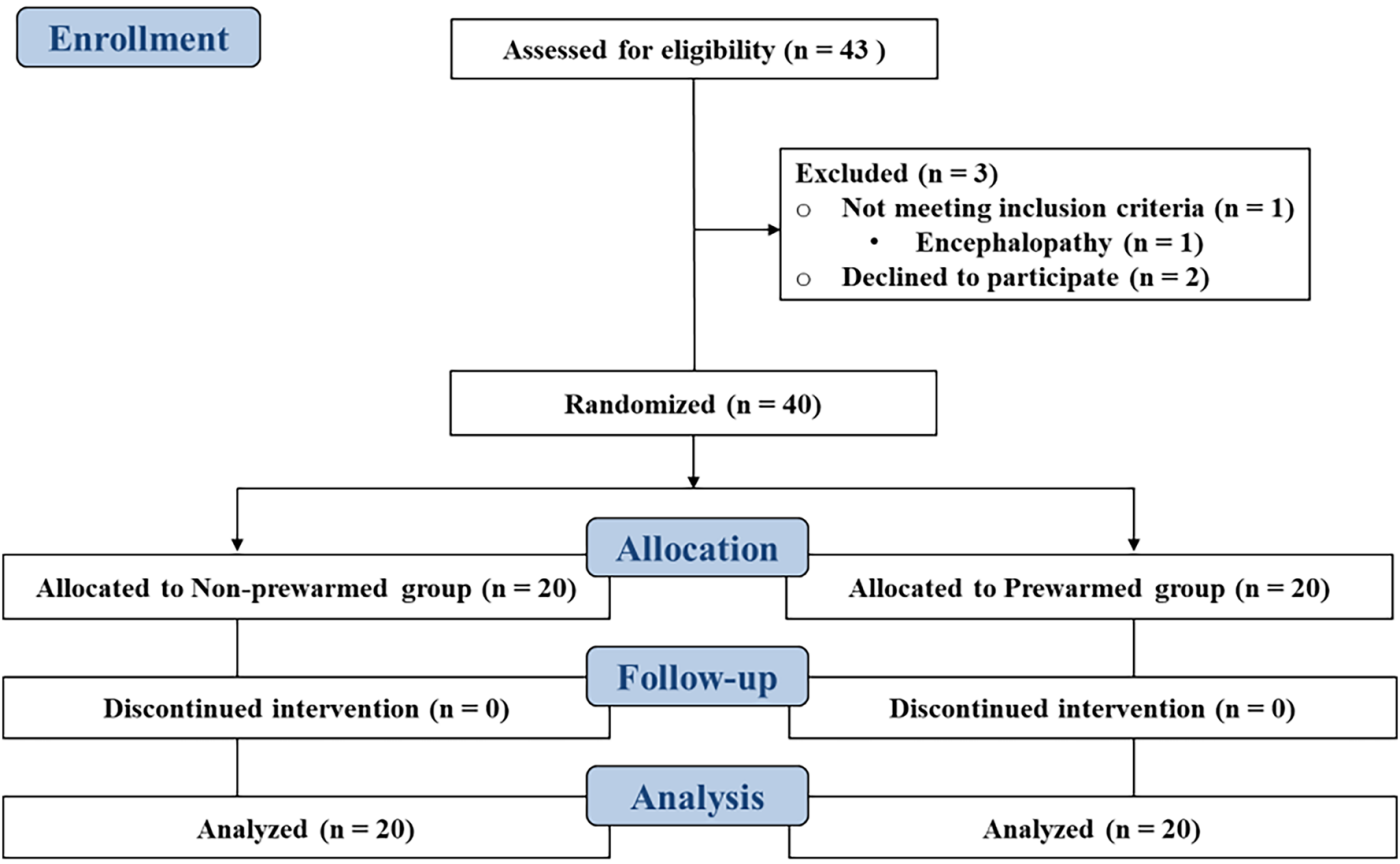
Consolidated standards of reporting trials (CONSORT) diagram.

As shown in Fig. 1, 40 adult patients (18–80 yr old) undergoing an elective living donor liver transplantation were enrolled from October 2018 to April 2019. Patients with preoperative fever (> 38.0 °C) or hypothermia (< 36.0 °C), previous transplant history, septic condition, encephalopathy, autonomic neuropathy, thyroid dysfunction, model for end-stage liver disease score > 30, and the risk of malignant hyperthermia were excluded from the study. This prospective, parallel-group, assessor-blind randomized controlled trial was approved by the Samsung Medical Center Institutional Review Board on July 04 2018 (SMC 2018-05-061-005) and registered at the Clinical Research Information Service on October 01 2018 (https://cris.nih.go.kr/, Identifier: KCT0003230**)**. Written informed consent was obtained from the recipients or their legal authorized representatives  and all methods were performed in accordance with the relevant guidelines and regulations.

### Intervention

Eligible patients were randomized 1:1 to either prewarming group or non-prewarming group using computer-generated numbers by a statistician not involved in patient screening or enrolment. Before entering the operating room, patients stayed at the preoperative waiting room for the intervention (prewarming vs. non-prewarming). The ambient temperature of the preoperative waiting room was thermostatically controlled being set at 25.0 °C at least 30 min before the patients' entrance. During the stay at the preoperative waiting room, non-invasive blood pressure, pulse oxymetry, and electrocardiography were monitored. Patients in prewarming group were covered from the neck to the feet by a disposable full-body forced-air blanket (Model 30000; 3M patient warming blanket, Eden Prairie, MN, USA). The blanket was tucked underneath the body to prevent direct leaking of forced-air to the ambient environment. They underwent forced-air warming during 30 min with the temperature of the forced-air being 43.0 °C using a dedicated warming device (Model 775; 3M Bair Hugger, St. Paul, MN, USA). Patients in non-prewarming group were covered by the same full-body forced-air blanket in the same way and a cotton blanket was additionally applied over it to prevent possible heat loss during the stay at the preoperative waiting room. They did not undergo active warming while the forced-air warming device was powered off. After the 30-min intervention, patients were immediately transferred to the operating room. The thermal comfort was assessed before and after the intervention in both groups with 11-point scale (0, worst imaginable cold; 5, identified thermal comfort; 10, worst imaginable hot)^17^. The thermal comfort change is the difference between the thermal comfort value before and after prewarming intervention.

### Standard thermal care

The ambient temperature of the operating room was thermostatically controlled being set at 24.0 °C at least 30 min before the patients’ entrance. A circulating water mattress (Blanketrol II, Clininnati Sub-Zero Products, Inc, Ohio) was placed on the operative table underneath the patient body being set at 37.0 °C. The patient’s body surface exposure was minimized with cotton blankets and surgical drapes throughout the anesthesia and surgery without intraoperative forced-air warming^23^. The upper extremities were additionally wrapped in vinyl to be separated from fluids outflowed from the surgical field. Active airway warming was performed using a heated wire breathing system (VentiMyst®, Flexicare Medical Limited, Mountain Ash, UK)^15^. A rapid fluid infusion device (Level 1® H-1200, Smiths medical, Dublin, OH, USA) was used to prevent hypothermia for all crystalloids, colloids, red blood cells, and fresh frozen plasma, while cryoprecipitate, platelets, and albumin products were infused at room temperature^24^. Warmed fluid was used to irrigate and wash the surgical field. When the patient’s core temperature reached to < 35.5 °C, the set temperature of operating room was changed to 26.0 °C and the set temperature of the circulating water mattress was changed to 40.0 °C. In contrast, when the patient’s core temperature reached to > 37.5 °C, the circulating water mattress and rapid fluid warming device were turned off.

### Monitoring and anesthetic induction

Anesthetic managements were performed according to the standardized institutional protocol, as described previously^25,26^. After initiating standard monitoring (pulse oximetry, 5-lead electrocardiography, and non-invasive arterial blood pressure measurements), anesthesia was induced with thiopental sodium (5 mg/kg) and maintained with isoflurane titrated to a bispectral index of 40–60. Mechanical ventilation was delivered at a tidal volume of 8 mL/kg (ideal body weight) and positive end-expiratory pressure of 6 mmHg using a mixture of medical air and oxygen at a fresh gas flow rate of 2 L/min, and the respiratory rate was adjusted as needed to maintain normocapnea. Direct arterial blood pressure monitoring was performed via the right radial artery and the right femoral artery. Central venous pressure was monitored through the right internal jugular vein and the right femoral vein. A large-bore 9-Fr catheter was placed in the right internal jugular vein in combination with a pulmonary arterial catheter (Swan-Ganz CCOmboV, Edward Lifesciences, LLC, Irvine, CA). The tip of the pulmonary arterial catheter was located at 1 cm proximal from the point where pulmonary artery occlusion occurred. During the arterial/venous line insertion, sterile drapes covered the body with minimal exposure of the skin to the ambient environment.

The routine intraoperative laboratory measurements included arterial blood gas analysis (including lactate), cell blood count, and coagulation profile (prothrombin time [PT], activated partial thromboplastin time [aPTT], and fibrinogen). They were regularly checked at the following time points: start of dissection phase (skin incision), start of the anhepatic phase, and 5/60/180 min after graft reperfusion, as described previously^11^.

### Data acquisition

In the preoperative operating room, core temperature was measured via the tympanic membrane using an infrared thermometer (Thermoscan 5, Braun, Kronberg, Germany) immediately before and after the intervention. In the operating room, core temperature was measured once via the tympanic membrane with an infrared thermometer (Thermoscan 5 IRT 4520, Braun, Kronberg, Germany) before the anesthesia was induced. After placing the pulmonary artery catheter, core temperature was continuously measured in the pulmonary artery and automatically recorded every 5 min into our electronic anesthesia record. The pulmonary catheter was removed immediately before transferring the patient to intensive care unit based on our center policy.

Other data were derived from electronic medical record system. Hemodynamic variables and ventilation-related variables were automatically recorded every 5 min during surgery into our electronic anesthesia record. The amount of intraoperative blood loss was calculated by using a formula designed for liver transplantation^27^. All perioperative laboratory findings were automatically recorded in the electronic medical record system.

### Variables and statistics

The primary outcomes were the incidence and duration of intraoperative hypothermia. Hypothermia was defined when core temperature was < 36.0 °C^28^. The sample size was calculated based on the previous study, demonstrating that 30-min prewarming before surgery reduced the intraoperative hypothermia incidence by 91.3%^29^. Given that liver transplant patients might be affected by relatively more thermal factors, we assumed that prewarming decreases intraoperative hypothermia by 85% and a minimum of 20 patients was required in each group (β = 0.8, α = 0.05, and 10% dropout rate). The secondary outcomes were the degree of core temperature change, blood lactate concentration, prothrombin time (PT [INR]), activated partial thromboplastin time (aPTT), and blood fibrinogen level as well as postoperative outcomes including 1-yr graft failure/mortality, acute kidney injury (AKI), major complication (the Clavien-Dindo grade was at least 2)^30^, and early graft dysfunction. Acute kidney injury was defined using the International Club of Ascites new classification for patients with cirrhosis^31^. Early graft dysfunction was defined as the presence of one or more of the followings: bilirubin ≥ 10 mg/dL on day 7, international normalized ratio ≥ 1.6 on day 7, and alanine or aspartate aminotransferases > 2000 IU/L within the first 7 days^32^. Continuous variables are expressed as mean ± standard deviation (SD) or median (interquartile range), being analyzed using student t-test or Mann–Whitney U test. Distribution normality was tested by Kolmogorov–Smirnov test. Paired data, such as core temperature before and after prewarming was analyzed using paired t-test. Repeatedly measured variables such as core temperature, prothrombin time, activated partial thromboplastin time, and fibrinogen level were analyzed using repeated measure ANOVA. Categorical variables are described as frequency (%), being analyzed using chi-square test or the Fisher’s exact test. A two-sided *P* value < 0.05 was considered statistically significant. All analyses were performed using SPSS 25.0 (IBM Corp., Chicago, IL, USA).

### Prior presentation

This study was presented in part at the 8th Annual Conference of the Korean Society of Transplantation Anesthesiologists, 20/03/2021.

## Results

The two groups were not significantly different regarding the baseline characteristics, anesthetic factors, and surgical factors (Table 1). Core temperature at the preoperative waiting room immediately before the intervention was not significantly different between the two groups (36.6 °C [36.5 °C, 36.8 °C] in non-prewarming group vs. 36.5 °C [36.4 °C, 36.7 °C] in prewarming group, *P* = 0.201). During the intervention, core temperature was not significantly changed in non-prewarming group (36.7 °C [36.4 °C, 36.8 °C] after the intervention, *P* = 0.741), whereas it was significantly increased in prewarming group (36.8 °C [36.6 °C, 37.0 °C] after the intervention, *P* = 0.018). Although, the degree of thermal comfort changed significantly greater in prewarming group than non-prewarming group (3 [1, 4] vs. 0 [0, 2], *P* < 0.001, Table 1), no patients requested adjustment in warming temperature or experienced thermal injury.

**Table 1:** Demographic and surgical factors of the patients undergoing liver transplantation.

	Non-prewarming group (*n* = 20)	Prewarming group (*n* = 20)	*P*
**Baseline characteristic**			
Age (yr)	55.3 ± 6.0	54.4 ± 10.0	0.718
Body Mass Index (kg/m^2^)	23.9 ± 3.0	25.4 ± 3.3	0.118
Body surface area (m^2^)	1.76 ± 0.15	1.75 ± 0.19	0.914
MELD score	14 ± 7	12 ± 6	0.345
Ascites (L)	0.9 ± 2.6	0.8 ± 1.7	0.865
Tc before prewarming (°C)	36.6 (36.5, 36.8)	36.5 (36.4, 36.7)	0.201
Thermal comfort change	0 (0, 2)	3 (1, 4)	< 0.001
Before prewarming	5 (5, 5)	5 (4, 5)	0.042
After prewarming	5 (4, 5)	8 (5, 8)	0.002
Thermal injury	0	0	
**Surgery Duration (min)**			
Post-induction phase	57 (49, 66)	59 (52, 71)	0.221
Dissection phase	155 (130, 161)	112 (97, 129)	< 0.001
Anhepatic phase	115 (96, 130)	117 (103, 139)	0.971
Reperfusion phase	209 (180, 239)	183 (170, 204)	0.068
Total surgery	434 ± 85	393 ± 91	0.153
Total anesthesia	530 ± 71	502 ± 88	0.276
**Intravenous fluid infusion (mL)**			
Crystalloid	5167 ± 2027	5140 ± 3147	0.974
Synthetic colloid	900 ± 308	965 ± 330	0.523
5% Albumin	887 ± 235	801 ± 284	0.301
**Surgical factors**			
Liver graft type (Right lobe)	19 (95.0)	19 (95.0)	> 0.99
Graft-to-recipient weight ratio	1.07 (0.99, 1.23)	1.06 (0.86, 1.20)	0.841
GV/SLV (%)	66.1 (51.9, 78.8)	56.9 (51.4, 67.0)	0.134
Estimated blood loss (L)	2.0 (1.4, 3.0)	1.8 (1.0, 4.0)	0.602

Data are presented as mean (standard deviation) or median (25th percentile, 75th percentile) or frequency (%). GV/SLV, graft volume to standard liver volume ratio; MELD, model for end-stage liver disease; Tc, core temperature.

**Figure 2:**
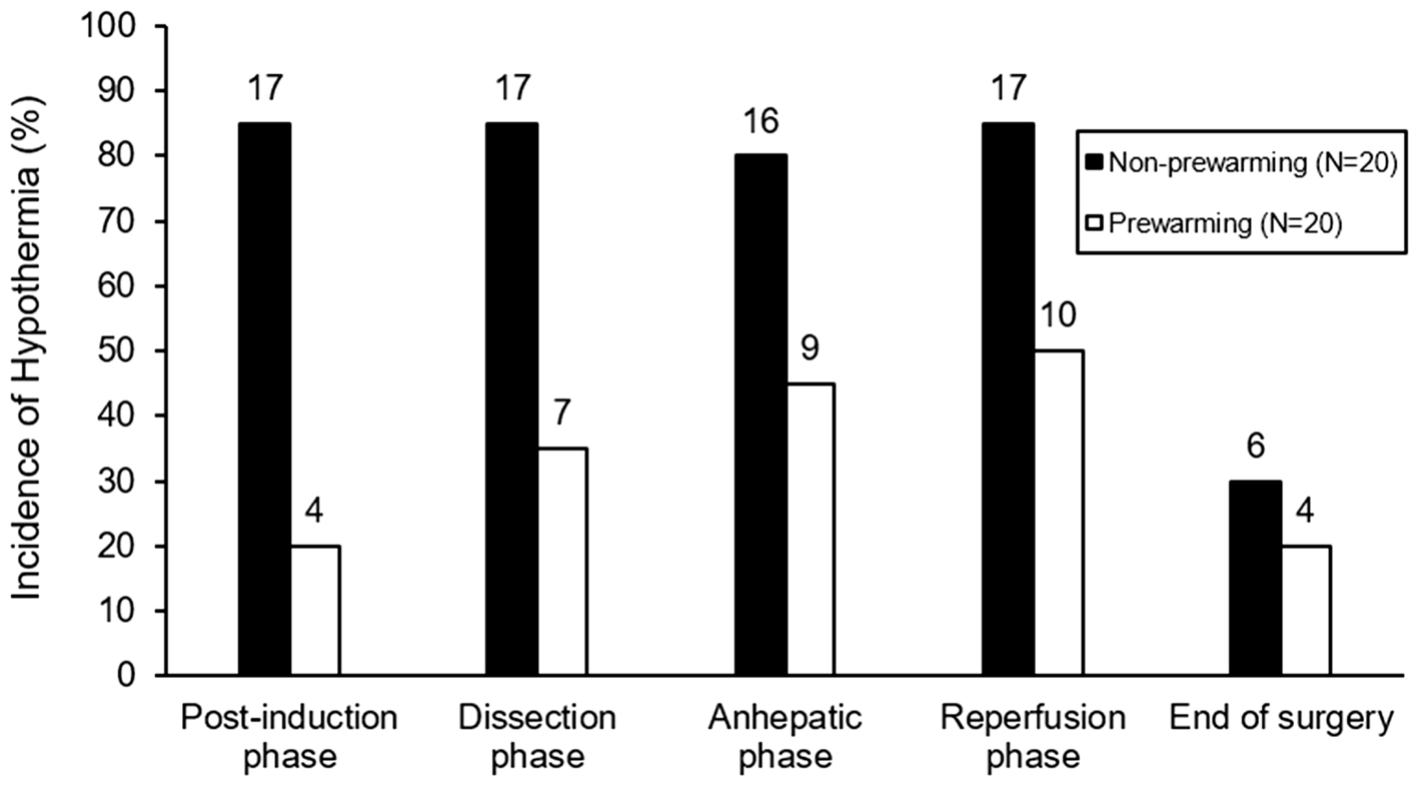
The proportion of patients with core hypothermia as the progress of anesthesia and surgery during liver transplantation. The number above the bars indicates frequency.

As shown in Fig. 2, intraoperative hypothermia incidence was significantly greater in non-prewarming group than in prewarming group (95.0% vs. 60.0%, odds ratio [OR] = 2.30 [1.39, 3.78], *P* = 0.020) and the duration of hypothermia was significantly longer in non-prewarming group (383 [108, 426] min vs. 60 [0, 221] min, *P* = 0.001). The difference between the initial core temperature at the entrance of operating room and the lowest intraoperative core temperature was significantly greater in non-prewarming group (1.4 °C [1.1 °C, 1.5 °C] vs. 0.9 °C [0.7 °C, 1.4 °C], *P* = 0.040).

**Figure 3:**
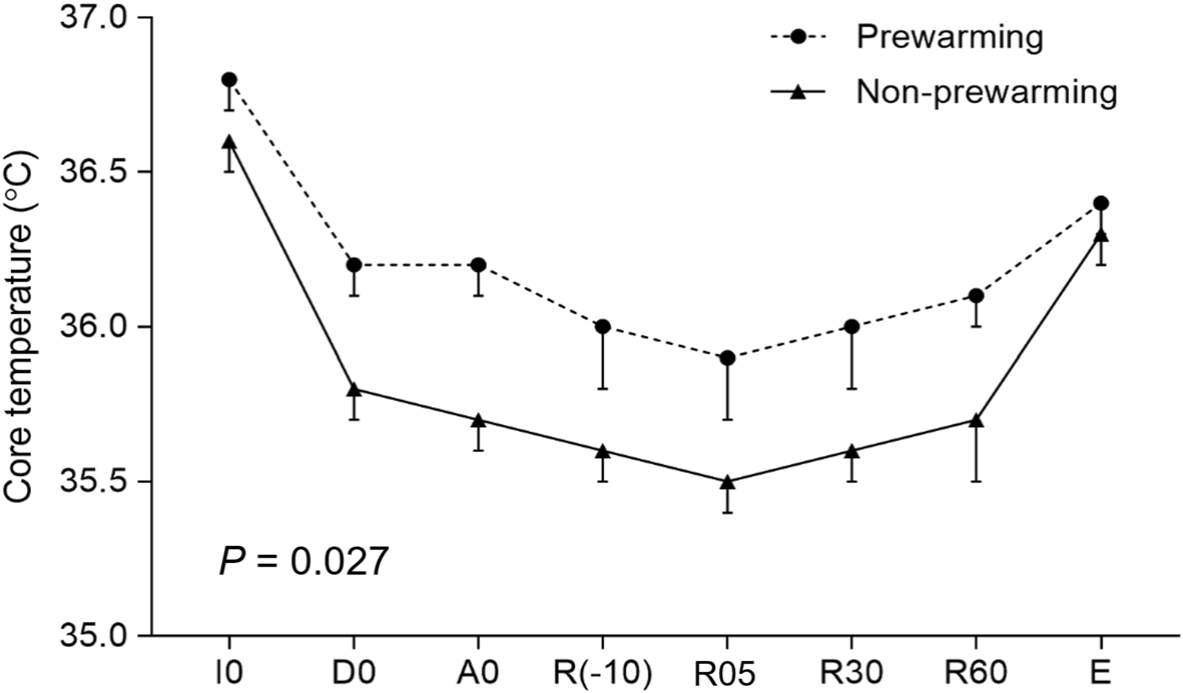
Serial change in intraoperative core temperature. Prewarming group showed higher core temperature at all analyzed times from the start of anesthetic induction to the end of surgery. The start of anesthetic induction [I0], start of dissection phase [D0], start of anhepatic phase [A0], 10 min before graft reperfusion [R(-10)], 5 min after reperfusion [R05], 30 min after reperfusion [R30], 60 min after reperfusion [R60], and at the end of surgery [E].

As shown in Fig. 3, core temperature dropped significantly during the post-induction phase (from anesthesia induction to the skin incision) in both groups (36.7 °C [36.4 °C, 36.8 °C] to 35.8 °C [35.6 °C, 35.9 °C], *P* < 0.001, in non-prewarming group; 36.9 °C [36.6 °C, 37.0 °C] to 36.2 °C [36.0 °C, 36.4 °C], *P* < 0.001, in prewarming group). The rate of core temperature drop during the post-induction phase was 0.8 (0.7, 1.0) °C/hr in non-prewarming group and 0.6 (0.3, 0.8) °C/hr in prewarming group (*P* = 0.019) while the duration of the post-induction phase was 57 (49, 66) min in non-prewarming group and 59 (52, 71) min in prewarming group. During the post-induction phase, hypothermia risk was significantly lower in prewarming group (20.0% vs. 85.0%, *P* < 0.001, Fig. 2). In contrast, the degree of change in core temperature was not significantly different between non-prewarming group and prewarming group during the dissection phase (0 °C [− 0.1 °C, 0.1 °C] vs. 0 °C [− 0.2 °C, 0.1 °C], *P* = 0.883), anhepatic phase (− 0.1 °C [− 0.2 °C, 0 °C] vs. − 0.1 °C [− 0.2 °C, 0 °C], *P* = 0.862), or post-reperfusion phase (0.2 °C [0.2 °C, 0.3 °C] vs. 0.2 °C [0.1 °C, 0.3 °C], *P* = 0.369) (Table 2), suggesting the particular impact of forced-air prewarming on the core-to-peripheral heat redistribution following anesthesia induction. Core temperatures repeatedly measured throughout the surgery were significantly higher in prewarming group (*P* = 0.027).

**Table 2:** Hypothermia duration and core temperature change during the indicated periods.

	Non-prewarming group(*n* = 20)	Prewarming group (*n* = 20)	*P*
**Intraoperative hypothermia duration (min)**^a^			
During whole anesthesia	383 (108, 426)	60 (0, 221)	0.002
Before graft reperfusion	236 (112, 278)	48 (0, 125)	< 0.001
After graft reperfusion	145 (41, 192)	21 (0, 126)	0.009
**Decrement of core temperature (°C)**			
Initial Tc—lowest Tc until anhepatic start	1.4 (1.1, 1.5)	0.8 (0.6, 1.4)	0.040
Initial Tc—lowest Tc during surgery	1.4 (1.1, 1.5)	0.9 (0.7, 1.4)	0.040
Initial Tc—Tc at surgery end	− 0.4 (− 0.7, 0.1)	− 0.4 (− 0.7, 0.0)	0.659
**Core temperature change rate (**°**C/hr)**			
During the post-induction phase	− 0.8 (− 1.0, − 0.7)	− 0.6 (− 0.8, − 0.3)	0.007
During the dissection phase	0.0 (− 0.1, 0.1)	0.0 (− 0.2, 0.1)	0.883
During the anhepatic phase	− 0.1 (− 0.2, 0.0)	− 0.1 (− 0.2, 0.0)	0.862
During the reperfusion phase	0.2 (0.2, 0.3)	0.2 (0.1, 0.3)	0.369

Data are presented as frequency (percent) or median (25th percentile, 75th percentile). Tc, core temperature.

^a^10 min after graft reperfusion was not counted.

**Figure 4:**
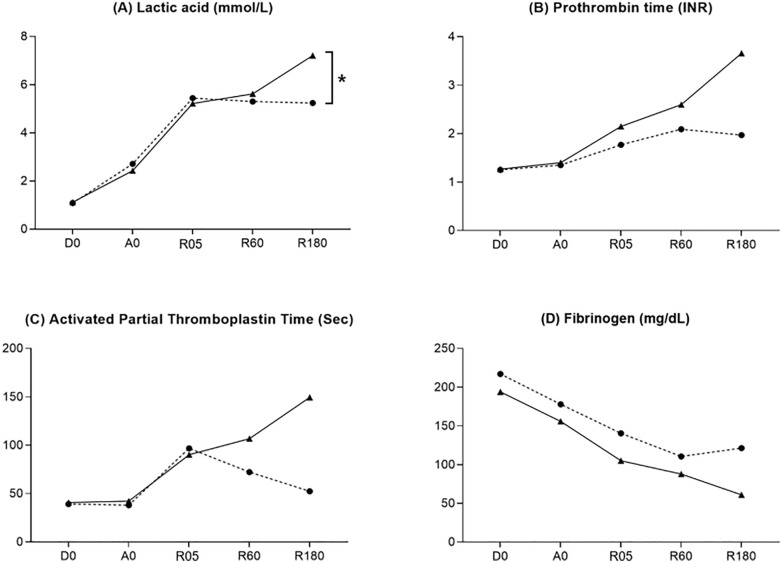
Changes of lactate and coagulation profile of non-prewarming group (straight line) and prewarming group (dotted line). The start of the dissection phase [D0], start of the anhepatic phase [A0], 5 min after graft reperfusion [R05], 60 min after graft reperfusion [R60], and 180 min after graft reperfusion [R180]. *Statistically significant (*P* < 0.05) between groups.

Blood lactate concentration continuously increased in non-prewarming group, whereas it decreased in prewarming group after graft reperfusion; accordingly, the degree of change in blood lactate concentration during the first 3 h after graft reperfusion was significantly different between the two groups (1.17 [3.31, 0.77] mmol/L in non-prewarming group and − 0.19 [0.13, − 0.48] mmol/L in prewarming group, *P* = 0.034, Fig. 4). Although statistical significance was not found, coagulation profile showed trend of recovery after graft reperfusion in prewarming group, whereas it did not recover in non-prewarming group (*P* = 0.192 in prothrombin time [INR], *P* = 0.323 in activated partial thromboplastin time, and *P* = 0.246 in Fibrinogen, Fig. 4). The two groups were not significantly different regarding blood loss (2000 [850, 2500] mL in non-prewarming group and 1350 [900, 3600] mL in prewarming group, *P* = 0.820) and red blood cell transfusion (1 [0, 3] units and 0 [0, 2] units, *P* = 0.698).

For postoperative clinical outcomes, the incidence of acute kidney injury (5.0% in non-prewarming group vs. 10.0% in prewarming group, *P* > 0.99), major complications (10.0% in both groups, *P* > 0.99), and early graft dysfunction (10.0% in both groups, *P* > 0.99) within first 7 days of liver transplantation were not significantly different between the two groups. Also, 1-yr graft failure risk (0 patients vs. 2 [10.0%] patients, *P* = 0.487) and 1-yr mortality (1 [5.0%] patients vs. 3 [15.0%] patients, *P* = 0.605) were not significantly different between non-prewarming group and prewarming group.

## Discussion

This is the first to test the effects of forced-air prewarming on intraoperative core temperature of liver transplant recipients who are at high risk of hypothermia and its complications. In this randomized clinical trial, 30-min forced-air prewarming significantly reduced the incidence and duration of intraoperative hypothermia. The thermic benefit of forced-air prewarming was mainly found during the post-induction phase before the skin incision when significant core-to-peripheral heat redistribution occurs. Forced-air prewarming increased core temperature already before the start of anesthesia, which was thought to be due to the increase in total body heat content, and also decreased the degree of core temperature drop immediately after the start of general anesthesia, suggesting the decrease in the amount of core-to-peripheral heat redistribution. Also, we found that patients undergoing forced-air prewarming experienced better biochemical courses after graft reperfusion (e.g. lactate and coagulation profile), indicating a better metabolic function of the newly implanted liver graft^9,10,12^. Although clinical impact of intraoperative hypothermia and the benefits of normothermia are well developed in various surgical settings^4,5,8^, they have not been well demonstrated in liver transplantation. Our findings add an important evidence on clinical benefits of intraoperative normothermia during liver transplantation and suggests incorporating forced-air prewarming into thermic strategy for liver transplantation.

In agreement with previous studies of other surgical settings^18,19^, we found that preoperatively delivered heat content using forced-air warming effectively prevents intraoperative hypothermia during liver transplantation. Prewarming increases heat content mainly in the peripheral compartment and decreases the core-to-peripheral temperature gradient^29,33^. The reduced temperature gradient between peripheral heat content and central heat content decreases core-to-peripheral heat flow following vasodilatation after anesthetic induction^33^. In our study, prewarming was conducted by forced-air warming device with a disposable full-body forced-air blanket^8^. In particular, full-body forced-air blanket is known to deliver about 95 watts of convection heat across the patient’s body surface when the forced-air warming device is set to 43.0 °C for 30 min^34^.

Generally, core temperature increases progressively after the post-induction core-to-peripheral heat redistribution^35^. However, disturbed hepatic thermogenesis with the end-stage liver disease, absence of hepatic heat production during the anhepatic phase, and the cold graft contributes to continual core heat loss until graft reperfusion as shown in the previous and current studies, making it hard to recover from hypothermia once it occurs^1,13^. Thus, it is more important to prevent the post-induction hypothermia which may be difficult to overcome until graft reperfusion when core heat can be regained following active hepatic heat production. Moreover, previous studies have demonstrated that the core-to-peripheral heat redistribution occurs regardless of intraoperative active warming^4,7^. Of importance, the effects of the alteration in the laminar air flow during surgery resulted from forced and warmed air on liver transplant patients, who are considered at high risk of immunomodulation, has not been evaluated^23^. Thus, forced-air prewarming should be considered as an important part of thermal strategy in liver transplantation regarding its efficacy and safety.

Clinical significance of intraoperative hypothermia has not been evaluated in liver transplantation. To our knowledge, there was only one study showing the relationship between intraoperative core temperature and post-transplant cytomegalovirus infection^36^, a surrogate indicator for immune dysfunction^37^. Thus, we evaluated clinical indicators, such as lactate and coagulation profiles, which represent sequential liver function during transplantation (particularly after graft reperfusion)^38,39^. About 70% of lactate clearance is dependent to the liver, which converts lactate to pyruvate through lactate dehydrogenoase^40^. Accordingly, impaired graft function or delayed liver regeneration after graft reperfusion decreases lactate clearance^38^. In our study, plasma lactate concentration started to decrease after graft reperfusion in prewarming group, whereas it continuously increased until 3 h after graft reperfusion in non-prewarming group. As mentioned above, liver graft performs vigorous quantitative/qualitative regeneration along with full metabolic functions; thus, the efforts to provide better thermal environment for liver cells are important for the critical time window^9,10,12^. Moreover, this effect would be more evident in patients who are with marginal grafts that has greater risk to fail to meet the recipient’s metabolic demands. Regarding coagulation profiles, it is well known that even mild hypothermia can result in coagulopathy by impairing the function of enzymes involved in the coagulation cascade^3,41^. That is, even mild hypothermia can increase the amount of intraoperative bleeding^3,6,7^.

In the current study, patients without prewarming did not recover blood fibrinogen level even after 3 h after graft reperfusion. Fibrinogen is synthesized in the liver and it is known that the newly transplanted liver is the source of ≥ 98% of the circulation fibrinogen after graft reperfusion^42^, indicating plasma fibrinogen as an indicator for early graft function and recovery.

Although the importance of thermal management is widely accepted, the reluctance to use prewarming in patients undergoing liver transplantation may be at least in part due to the lack of evidence of the effective and safe duration of prewarming. In our study, we demonstrated that prewarming only for 30 min with a widely used forced-air warming device effectively decreases the core-to-peripheral heat redistribution and prevents intraoperative hypothermia without side effects, being in line with studies of other surgical populations^20^. Moreover, the effects of prewarming was questioned in liver transplant recipients because their peripheral vascular heat barrier could be thought to be already disturbed based on hyper-dynamic circulation and decreased systemic vascular resistance^21,22,25^. The current study is the first to prove the efficacy and safety of forced-air prewarming even for liver transplant recipient with end-stage liver disease or cirrhotic circulatory changes.

This study has limitations. First, patients who underwent prewarming might have been aware of the intervention. However, it is unlikely that this influenced the results because patients cannot control core heat content or core temperature irrespective of the awareness. Also, data collection was done by blinded assessors. Second, the forced-air prewarming strategy used in the current study could not be implicated in deceased donor liver transplantation at which most patients are critically ill, and many body parts should be directly observed and easily approached. In such situations, forced-air warming using a cover type blanket is not feasible although an underbody type blanket could be an alternate. Third, there was possibility of type II error for secondary outcomes. Further research with sufficient sample size is warranted to analyze the effects of prewarming on clinical outcomes.

In the current study, we found that 30-min forced-air prewarming is effective to reduce post-induction core heat loss, prevent intraoperative hypothermia, and decrease the duration of intraoperative hypothermia in living donor liver transplantation. Also, lactate clearance early after graft reperfusion was improved in relation to the use of forced-air prewarming and consequent greater core temperature during the reperfusion phase. No clinically significant side effects were found with forced-air prewarming. Therefore, we concluded that forced-air prewarming is an effective and safe method to prevent intraoperative hypothermia with potential clinical benefits in living donor liver transplantation.

## Data Availability

The data that support the findings of this study are available on request from the corresponding author. The data are not publicly available due to privacy or ethical restrictions.
